# Histological Evaluation of Sodium Iodide-Based Root Canal Filling Materials in Canine Teeth

**DOI:** 10.3390/ma17246082

**Published:** 2024-12-12

**Authors:** Jae Hee Lee, Sak Lee, Hye-shin Park, Yu-Jin Kim, Hae-Hyoung Lee, Mi-Ran Han, Jun-Haeng Lee, Jong-Bin Kim, Ji-Sun Shin, Jong-Soo Kim, Jung-Hwan Lee

**Affiliations:** 1 Department of Pediatric Dentistry, College of Dentistry, Dankook University, 119 Dandae-ro, Cheonan 31116, Republic of Korea; 12140556@dankook.ac.kr (J.H.L.); gptls1209@naver.com (H.-s.P.); miraneee@dankook.ac.kr (M.-R.H.); haeng119@naver.com (J.-H.L.); jbkim0222@dankook.ac.kr (J.-B.K.); pedoshin@dankook.ac.kr (J.-S.S.); 2 Department of Oral Pathology, Seoul National University Dental Hospital, Seoul 03080, Republic of Korea; leesak@snu.ac.kr; 3 Department of Oral Pathology, School of Dentistry, Seoul National University, Seoul 03080, Republic of Korea; 4 Institute of Tissue Regeneration Engineering (ITREN), Dankook University, 119 Dandae-ro, Cheonan 31116, Republic of Korea; yujin10316426@gmail.com (Y.-J.K.); haelee@dku.edu (H.-H.L.); 5 Department of Biomaterials Science, College of Dentistry, Dankook University, 119 Dandae-ro, Cheonan 31116, Republic of Korea; 6 Department of Nanobiomedical Science & BK21 PLUS NBM Global Research Center for Regenerative Medicine, Dankook University, 119 Dandae-ro, Cheonan 31116, Republic of Korea; 7 UCL Eastman-Korea Dental Medicine Innovation Centre, Dankook University, 119 Dandae-ro, Cheonan 31116, Republic of Korea; 8 Cell & Matter Institute, Dankook University, 119 Dandae-ro, Cheonan 31116, Republic of Korea; 9 Mechanobiology Dental Medicine Research Center, Dankook University, 119 Dand-ro, Cheonan 31116, Republic of Korea

**Keywords:** sodium iodide, NaI, root canal filling material, biocompatibility test, canine teeth study

## Abstract

A novel water-soluble root canal filling material based on sodium iodide (NaI) has been developed to overcome the limitations of existing iodine-based formulations. However, the biological stability of this approach in animal studies remains unverified. This study evaluated the biocompatibility of NaI compared to commercial root canal filling materials (Calcipex II and Vitapex^®^) in pulpectomized canine teeth to assess its clinical applicability. Following a four-week observation period, none of the experimental groups exhibited tooth mobility or fistula formation. Radiographic and micro-CT analyses revealed no radiolucency in periapical lesions. Histopathologic evaluation demonstrated the absence of inflammatory responses in periapical regions across all material groups, with histological inflammation scoring 0. High-magnification histological examination of periapical areas showed well-preserved periodontal ligament tissue in all groups. Despite certain limitations of NaI-based fillings in the pulp cavity, including loss of radiopacity and tooth discoloration, NaI demonstrates potential as a safe and effective alternative for pulp filling material, particularly due to its minimal risk of root resorption and inflammatory response.

## 1. Introduction

Extensive clinical evidence has demonstrated that endodontic therapy in deciduous dentition can lead to premature tooth loss due to accelerated root resorption processes [1,2,3,4,5]. Recent investigations have revealed significant concerns with iodoform-containing materials (such as Vitapex^®^): enhanced cytotoxicity in cellular models [6], increased root resorption patterns compared to alternative materials [7], and accelerated resorption rates in treated teeth versus untreated counterparts [8]. Altogether, there is a need to develop new root canal filling materials to tackle the above drawbacks. The collective findings underscore the critical need to develop novel root canal filling materials that can overcome these biological limitations and provide more predictable clinical outcomes [9].

Vitapex^®^, a widely used iodoform-containing material, consists of calcium hydroxide, iodoform, silicone oil, and proprietary components [10]. Given the established link between iodoform and root resorption [7], our research focused on identifying alternative components while maintaining the enhanced antibacterial effects known to occur with calcium hydroxide combinations [11]. Sodium iodide, commonly used in thyroid cancer treatments, emerged as a promising candidate due to its proven biocompatibility [12]. Initial development by Choi et al. [13] led to the successful formulation of NaI, a novel material where sodium iodide replaced iodoform in a calcium hydroxide and silicone oil matrix. Their study demonstrated that sodium iodide-based materials significantly reduced osteoclastogenic activity through downregulation of key markers including TRAP, cathepsin K, NFATc1, and c-Fos, suggesting a lower root resorption potential compared to iodoform-based materials [14,15]. Subsequent work by Chang et al. focused on improving injectability using low-density silicone oil, however, the resulting formulation exceeded ISO 6876 solubility standards, limiting its clinical applicability [16]. To address this limitation, the next study incorporated lanolin as a safe supplement to decrease the solubility and finalized the optimization of all ingredients of the sodium iodide-based filling material to meet ISO standards [17,18,19].

This study sought to assess the biocompatibility of our optimized sodium iodide-based formulation through a comparative analysis with established commercial root canal filling materials in pulpectomized canine teeth under clinically sterile conditions. Two gold-standard materials were selected for comparison—Vitapex^®^, representing traditional iodoform-containing materials, and Calcipex II, exemplifying calcium hydroxide-based formulations—to evaluate the clinical viability of our novel approach.

## 2. Material and Methods

### 2.1. Preparation for Sample

In this study, calcium hydroxide (Sigma-Aldrich, Burlington, MA, USA), sodium iodide (AlfaAesar, Heysham, UK), low-density silicone oil (Shin-Etsu Silicone KF-96 350 cst, Shin-Etsu Chemical Co., Tokyo, Japan), and lanolin (Deajung, Gyeonggi-do, Republic of Korea) for solubility adjustment were employed. The injectable pastes were prepared by blending the components on a glass surface using a spatula under sterile conditions. The composition of all experimental groups was 28.75:28.75:40:2.5 wt% for calcium hydroxide, odium iodide, low-density silicone oil, and lanolin, meeting ISO physicochemical standards [17,19]. As commercially available control materials, Calcipex II (Techno-Dent, Bangkok, Thailand) and Vitapex^®^ (Neo Dental, Tokyo, Japan) were chosen. Following root canal treatment (pulpectomy), root-end filling material was randomly applied. Cavities were restored with IRM^®^ (Dentsply Sirona, York, PA, USA) and sealed with 3M™ Filtek™ Supreme Flowable Restorative (3M EPSE, Seefeld, Germany) according to the manufacturer’s instructions. As a positive control to reveal the inflammatory response, 100 µL of 10^8^ CFU/mL Enterococcus faecalis (*E. faecalis*, ATCC 19433, Manassas, VA, USA) was injected without root filling materials.

### 2.2. Animal Study

**Figure 1:**
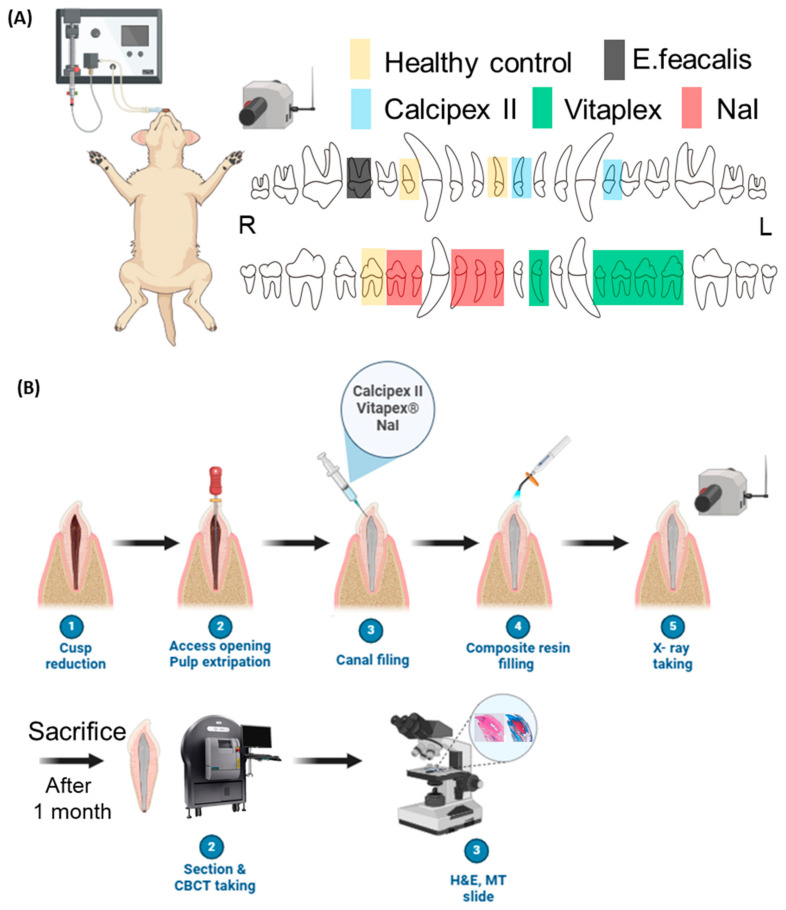
Summary of dog animal study investigating biocompatibility of newly developed root canal filling material in healthy root canal after aseptic pulpectomy. (**A**) Tooth treatment plan for root canal filling materials, healthy control, and *E. faecalis* positive control. (**B**) One-visit pulpectomy and filling material protocols. After 1 month, dog was scarified for histological analysis.

This animal study was approved by the Institutional Animal Care and Use Committee (CRONEX-IACUC: 202312001) and conducted following the biological safety evaluation protocol in accordance with ISO 7405 [20]. A male mongrel dog (12 months old, 40 kg) was selected for this study. Healthy maxillary and mandibular incisors and premolars with complete root formation were chosen. After visual and radiographic examination, 16 teeth without periapical inflammation or mobility were initially allocated to different groups for root-end filling material restoration, followed by visual, radiographic, and histological evaluations (Figure 1A).

General anesthesia was administered by intramuscular injection of a Zoletil 50 (Virbac S.A., Carros, France) and Rompun (Xylazine Hydrochloride, Bayer, Leverkusen, Germany) mixture (1:1, 0.2 mL/kg). After securing the airway using a laryngoscope, a 7.0 intubation tube was inserted, and inhalation anesthesia was maintained with an isoflurane (Piramal Critical Care, Inc., Mumbai, India) and oxygen mixture (2:1).

Following jaw fixation, full-mouth photographs and radiographs were taken. The oral cavity was cleaned with hydrogen peroxide and saline solution, followed by local anesthesia with 2% lidocaine and tooth isolation using cotton rolls. Cusps were removed using a #256R diamond bur, the pulp chamber was accessed using a #330 carbide bur, and pulp was extirpated using a barbed broach. Root canals were prepared using hand K-files and NiTi rotary files with saline irrigation. Canals were dried with sterile paper points and randomly assigned to the control, Calcipex II, Vitapex^®^, or NaI group for obturation following the manufacturer’s instructions (Neo Dental International, Inc., Federal Way, WA, USA). Final restoration was performed using IRM^®^ and composite resin, followed by intraoral and radiographic documentation. The animal condition was monitored regularly by a veterinarian, with follow-up examinations at 1 and 4 weeks post-obturation.

After 4 weeks, the animal was euthanized using Succipharm (Suxamethonium Chloride Hydrate 50 mg, Komipharm, Seoul, Republic of Korea) following general anesthesia. Teeth were harvested with surrounding bone and fixed in 10% formalin (Duksan Pure Chemicals, Ansan, Republic of Korea). Block specimens were analyzed using Micro-CT (Quantum FX uCT; Perkin Elmer, Hopkinton, MA, USA) for 3D imaging. Specimens were decalcified in 14% EDTA (pH 7.0) for approximately 3 months, with the decalcification solution changed each week. After decalcification, the specimens were processed overnight according to a routine procedure using graded ethanol and a xylene mixture for dehydration. They were then embedded in paraffin wax (327204, Sigma Chemical, USA). We cut 3–4 μm thick sections and used those on poly-L-lysine-coated glass slides (5116-20F, Muto Pure Chemicals Co., Ltd., Tokyo, Japan).

Deparaffinized slides were stained with hematoxylin and eosin. Hematoxylin (GHS332, Sigma Chemical, St. Louis, MO, USA) for 10 min in tap water was followed for 2 min by eosin (318906, Sigma Chemical, USA). The, the slides were stained using Masson’s trichrome staining kit (HT15-1KT, Sigma-Aldrich, St. Louis, MO, USA). Histological images were obtained using an optical microscope (BX50; Olympus, Tokyo, Japan) and automated slide scanner (TW-SM01; TaeWoong Medical Co. Ltd., Goyang, Republic of Korea). Histopathological evaluation was performed by a pathologist according to the criteria in Table 1 (Figure 1B).

Immunohistochemistry was performed according to the conventional protocol, where 1:100 of primary antibody (1:200; ab205719, Abcam, 1:200; ab205718, Abcam, Cambridge, UK) of CD68 (orb388934, Biorbyt, Cambridge, UK) and CD45 (5788-MSM5-P1ABX, Thermofisher, Walthan, MA, USA) and 1:200 of secondary antibody were used (Table 1) [21,22,23].

The slides were examined via light microscopy by an oral pathologist blinded to the treatment groups. Immunohistochemical results were referenced to accurately identify inflammatory cells such as lymphocytes, macrophages, and polymorphonuclear neutrophils. The number of inflammatory cells (mononuclear and polymorphonuclear) was determined in three representative fields at 400× magnification, measured in the periapical periodontal ligament area. Data were analyzed using the Kruskal–Wallis test followed by Dunn’s test (α = 0.05)

### 2.3. Radiopacity Test in Endodontic-Treated Resin Tooth

To assess the radiopacity of root canal filling materials under different conditions, artificial root canal treatments were conducted on 12 mandibular premolar resin teeth models. The working length was established using diagnostic radiography, with a #15 K-file inserted into the root canal until its tip appeared 1 mm short of the radiographic apex. Root canals were shaped using K-files up to size #35, utilizing normal saline and sodium hypochlorite solutions for irrigation [19,24]. Following canal drying with paper points, an injectable paste was administered via syringes until extrusion through the canal orifice occurred, after which the syringes were removed. The access cavities were filled with the same injectable paste to ensure uniform evaluation [16]. Periapical radiographs were obtained post-operatively to verify the root canal filling quality. The materials’ radiography was evaluated after a 1-month exposure to air and distilled water at room temperature (30–50% relative humidity). An aluminum step wedge (50 mm length, 20 mm width) with a minimum 98% aluminum purity, maximum 0.1% copper, and 1.05% iron content was utilized alongside the specimen-filled teeth. These were positioned on Kodak Insight X-ray film (Rochester, NY, USA). Radiographic images were captured using a Kodak-2200 X-ray machine (Kodak Insight, Rochester, NY, USA) with parameters set at 7 mA current, 70 kV voltage, 0.3 s exposure time, and 300 mm source-to-film distance. ImageJ version 1.53a grayscale software (National Institutes of Health, Bethesda, MD, USA) was employed to analyze the X-ray images. The specimens’ radiopacity was evaluated against various thickness steps of the aluminum wedge.

### 2.4. Statistical Analysis

The data were presented as mean ± standard deviation (SD) values and analyzed using SPSS software version 26.0 (SPSS Inc., Chicago, IL, USA). The Kruskal–Wallis test, along with Dunnett’s multiple comparison test, was employed to evaluate the distribution of data within groups. The level of significance was set at α = 0.05, and the notation ‘ns’ was used to indicate no statistical significance.

## 3. Results

### 3.1. Radiographic and Micro-Computed Tomographic Evaluation

**Figure 2:**
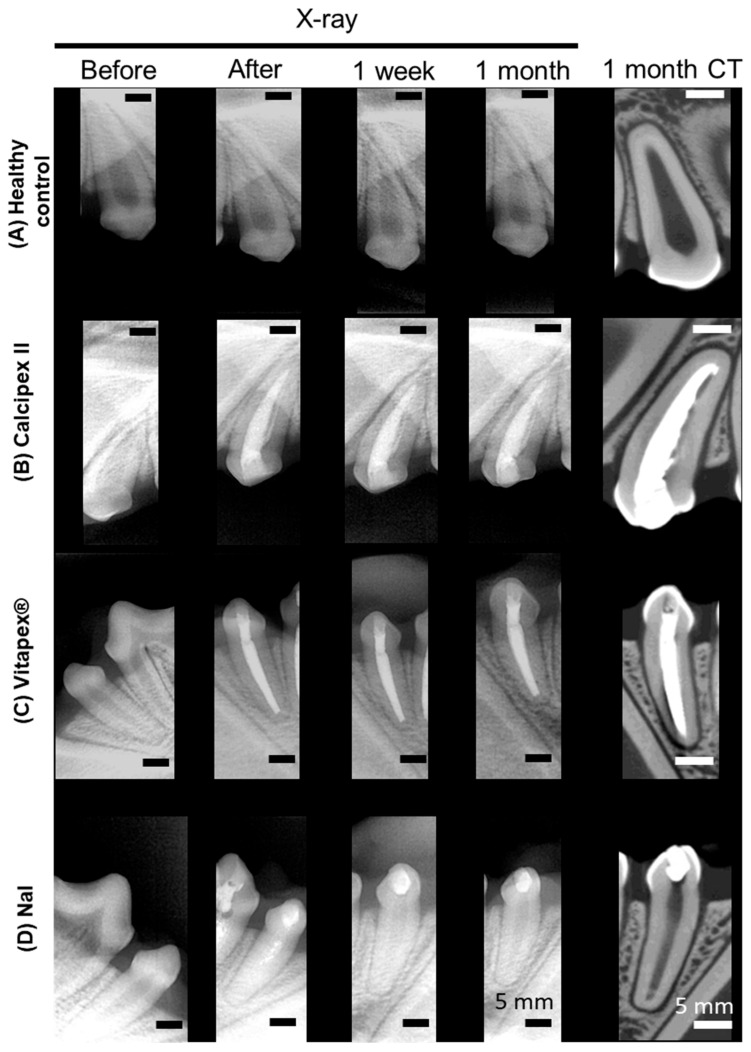
Representative before and after treatment X-ray and CT images of mongrel dog teeth following pulpectomy and subsequent filling with different root canal filling materials. (**A**) Control group without pulpectomy, showing normal pulp tissue. (**B**) Tooth filled with Calcipex II. (**C**) Tooth filled with Vitapex^®^. (**D**) Tooth filled with NaI. No prominent changes were observed in the periapical region and the periodontium in any groups from X-ray and CT. White areas in the pulp chamber in Calcipex II and Vitapex^®^ indicate continuous radiopacity of filled materials, while NaI gradually lost its radiopacity.

Sixteen canine teeth were evaluated throughout the experimental period, with all specimens maintaining structural integrity of their coronal composite resin restorations. Clinical assessment at 4 weeks post-operatively revealed no evidence of tooth mobility or sinus tract formation on gum tissues. Periapical radiographic examination demonstrated the absence of pathological radiolucencies in all specimens (Figure 2). However, the NaI-based material exhibited a progressive loss of radiographic contrast at one week post-obturation, attributed to the inherent dissolution characteristics of NaI leading to loss of radiopacity (Supplementary Figure S1), while maintaining volumetric stability of the filling material.

High-resolution micro-computed tomographic analysis at the 4-week endpoint enabled three-dimensional assessment of the periapical region, revealing no evidence of pathological osseous changes or periradicular radiolucencies across all experimental groups (Figure 2).

### 3.2. Histological Analysis

**Figure 3:**
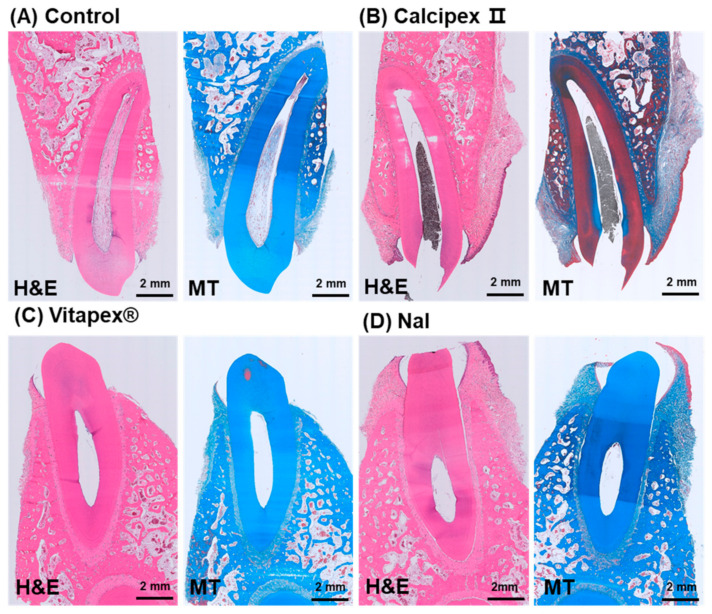
Representative low-magnification histological images of mongrel dog teeth following pulpectomy and subsequent filling with different root canal filling materials. (**A**) Control group without pulpectomy, showing normal pulp tissue. (**B**) Tooth filled with Calcipex II, showing the area where the pulp was removed and is now filled with Calcipex II. (**C**) Tooth filled with Vitapex^®^. (**D**) Tooth filled with NaI. Vitapex^®^ and NaI were washed out during the decalcification step. Only Calcipex II remained in the pulp chamber. H&E, hematoxylin and eosin staining; MT, Masson’s trichrome. No prominent inflammatory or pathological changes were observed in the periapical region and the periodontium in any groups. No pathological collagen composition was detected either. Masson’s trichrome staining was used to highlight the periodontal ligament area, emphasizing the abundant collagen present within it. The *E. faecalis* group showed inflammatory signs as a positive control (see Supplementary Figure S2).

At a low magnification, no distinct expansion of the periapical PDL space was observed in the four groups, excluding the *E. faecalis* group, and no other specific pathological findings were noted (Figure 3). In the *E. faecalis* group, no distinct expansion of the periapical PDL space was observed. However, in MT staining, the staining intensity in the periapical PDL space appeared lighter compared to the other groups (Supplementary Figure S2B).

**Figure 4:**
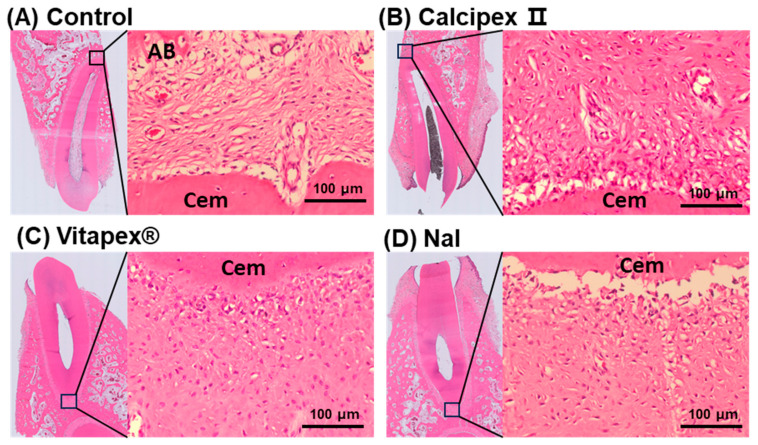
Representative high-magnification histological images (H&E) focusing on the periapical region of mongrel dog teeth after pulpectomy with different root canal filling materials. (**A**) Control group, showing normal periapical periodontal ligament (PDL) tissue composed of fibrocollagenous tissue and a few blood vessels, indicating healthy periapical conditions with no evidence of inflammatory infiltration or other pathological changes. (**B**–**D**) Teeth filled with Calcipex, Vitapex, and NaI after pulpectomy, respectively, showing periapical regions with fibrocollagenous tissue and small blood vessels, with no significant pathological differences compared to the control group (**A**). NaI groups showed similar a normal periapical tissue structure. Cem, cementum; AB, alveolar bone; H&E, hematoxylin and eosin staining. The *E. faecalis* group showed inflammatory signs as a positive control (see Supplementary Figure S2).

At a high magnification, the periapical PDL space of the four groups, excluding the *E. faecalis* group, was composed of fibroblasts, cementoblasts, osteoblasts, osteoclasts, and mesenchymal stem cells within fibrous connective tissue typically observed in normal PDL (Figure 4). Only a very small number of inflammatory cells, such as lymphocytes, mast cells, eosinophils, macrophages, and neutrophils, were observed (Figure 4). However, in the *E. faecalis* group, a marked increase in inflammatory cells such as neutrophils, lymphocytes, and macrophages was observed compared to the other four groups (Supplementary Figure S2C).

**Figure 5:**
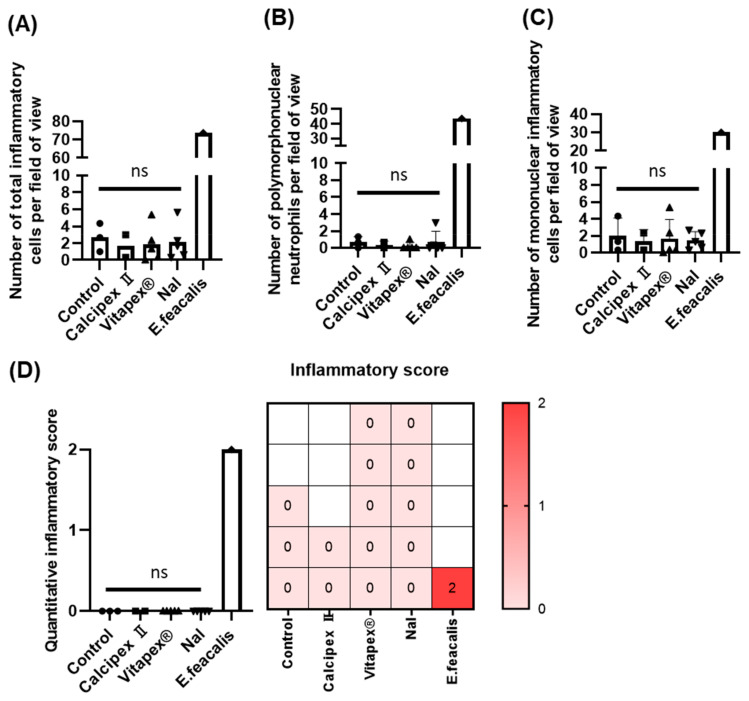
Quantitative evaluation of periapical inflammation after applying root canal filling materials based on H&E-stained images. (**A**) Total number of inflammatory cells in the periapical area. (**B**) Number of neutrophils. (**C**) Number of mononuclear inflammatory cells. (**D**) Quantitative inflammatory scoring based on histology images (0, mild inflammation; 1, moderate inflammation; 2, severe inflammation). There was no significant difference in the number of inflammatory cells between the control group and all filling material groups, including NaI. However, when compared with the *E. faecalis* group, there were significant differences in all experimental groups, including the control group. Non-parametric Kruskal–Wallis test with Dunn’s multiple comparison test was performed. No significant (NS) is based on *p* value at 0.05. Different shapes from bar graph mean different groups.

In the quantitative evaluation of inflammatory cells in the apical region, the control groups, the Nal group, Vitapex group, and Calcipex group showed very low numbers without significant differences among them (Figure 5A). The number of polymorphonuclear neutrophils, which are involved in acute inflammatory responses, also showed no significant differences across the four groups, excluding the *E. faecalis* group (Figure 5B). Similarly, the number of lymphocytes and macrophages, which are involved in chronic inflammatory responses, showed no significant differences among these groups (Figure 5C). On the other hand, when comparing the *E. faecalis* group with the other four groups, there was a significant difference in the number of polymorphonuclear and mononuclear inflammatory cells (Figure 5A–C).

To support the quantitative evaluation of inflammatory cells observed in high-magnification H&E slides, immunohistochemistry was performed. CD68 staining was used to specifically identify macrophages, while CD45 staining was used to identify pan-leukocyte markers including lymphocytes, macrophages, mast cells, and plasma cells.

**Figure 6:**
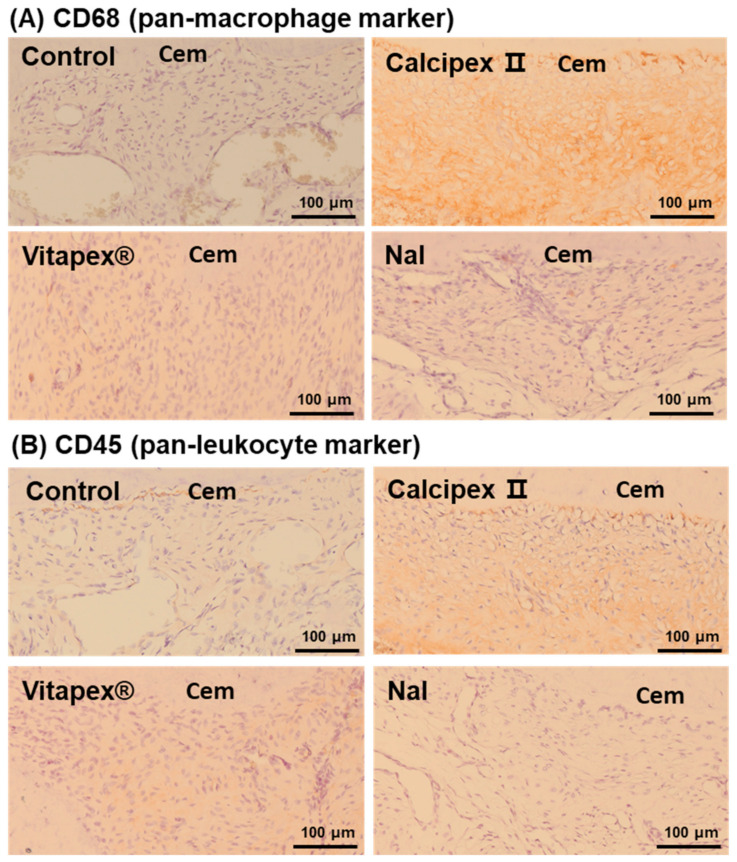
Representative immunohistochemical staining of CD68 and CD45. Pan macrophage marker and leukocyte marker were used to investigate apical lesion inflammation after applying root canal filling materials, respectively. Immunohistochemical staining of (**A**) CD68 and (**B**) CD45. There was no significant difference in the number of inflammatory cells between the control healthy group and any filling material groups, including NaI. However, when compared with the *E. faecalis* positive control group, there were significant differences in all experimental groups, including the control group.

In the four groups, including the Nal group, CD68-positive macrophages and CD45-positive inflammatory cells were scarcely observed except the E. feacalis group (Figure 6A,B). However, in the *E. faecalis* group, numerous CD68-positive macrophages and CD45-positive inflammatory cells were observed, consistent with the results from the inflammatory cell count evaluation on H&E slides (Supplementary Figure S2D,E).

## 4. Discussion

The evolution of root canal filling materials for primary teeth reflects continuous efforts to optimize clinical outcomes. Zinc oxide eugenol (ZOE), while traditionally used, showed limitations including slow resorption rates, periapical tissue irritation, and potential adverse effects on permanent tooth buds [25,26,27,28,29]. The introduction of calcium hydroxide in 1920 marked a significant advancement [11], further enhanced by the addition of iodoform, which improved antibacterial efficacy and resorption characteristics [30]. This combination led to the development of successful commercial products like Vitapex^®^, achieving success rates of 84–100% [31,32,33,34]. However, the observed acceleration of root resorption with iodoform-containing materials prompted our development of a sodium iodide-based alternative, aiming to maintain the benefits of iodine while minimizing undesirable effects on root stability [7,13,16,19].

The biocompatibility assessment of new dental materials in healthy conditions before adjustment in a clinically relevant microenvironment represents a crucial initial step in their development pathway. Our study utilized a canine model, which offers advantages in evaluating dental materials due to similarities in tooth structure and healing processes with human teeth [35]. The careful selection of healthy teeth and standardized pulpectomy procedures provided a controlled environment to assess the material’s interaction with periapical tissues. This approach aligns with ISO guidelines for biological evaluation of dental materials, establishing a foundation for understanding the material’s basic safety profile before investigating its efficacy in pathological conditions [20]. The four-week observation period, while sufficient for initial biocompatibility assessment, represents a limitation in evaluating long-term material performance. This timeframe was chosen based on ISO 7405 guidelines for preliminary biological evaluation and allows observation of acute tissue responses and early integration. However, future studies should incorporate extended observation periods of 3–12 months to assess potential delayed effects such as material degradation, chronic tissue responses, and impacts on permanent tooth development. Such long-term studies would be particularly relevant given the gradual loss of radiopacity observed with NaI-based materials and the need to understand its clinical implications over time.

To objectively and accurately detect inflammatory cells, immunohistochemistry for CD68 and CD45 was referenced. CD68 is a marker specific to macrophages, while CD45 is a cell surface marker expressed on inflammatory cells such as lymphocytes, eosinophils, basophils, macrophages, mast cells, and plasma cells. In evaluating inflammatory cells using CD45 and CD68, both the morphology of cells observed in H&E staining and the cytoplasmic positivity by each antibody were considered. Interestingly, CD45-positive and CD68-positive cells are observed at the outermost part of the cementum, between the cementum and the periodontal ligament (PDL) (CD45 and CD68 for Calcipex II group in Figure 6), which are thought to be cementoblasts rather than inflammatory cells.

The comparative analysis between our NaI-based material and commercial products (Calcipex II and Vitapex^®^) demonstrated equivalent safety profiles in healthy pulpectomized teeth. Particularly noteworthy was the inclusion of *E. faecalis* as a positive control for the inflammatory response, which provided a clear contrast to validate our histological and immunological findings. While this study’s limitation to a single dog with selected teeth might raise questions about its statistical power, this design reflects our commitment to the 3R principle (Reduction, Refinement, Replacement) in animal research. The absence of significant inflammatory responses across all treatment groups supports the biological safety of our NaI formulation, though future studies will need to assess its performance in infected root canals to fully establish its clinical potential.

The observed limitations of the NaI-based material, particularly its progressive loss of radiopacity and potential for tooth discoloration, warrant further material optimization. Future development should focus on incorporating radiopaque agents (i.e., ZnO_2_ or TiO_2_ particles) with greater stability or exploring alternative iodine-containing compounds that maintain radiographic visibility while preserving the material’s biological advantages. Additionally, the incorporation of color-stable components or protective barriers might help address aesthetic concerns. These modifications should be carefully balanced against the material’s current favorable biological profile and physical properties. Clinical protocols might also need adaptation, such as regular radiographic monitoring schedules or specific placement techniques, to compensate for these limitations while maximizing the material’s therapeutic benefits.

## 5. Conclusions

**Figure 7:**
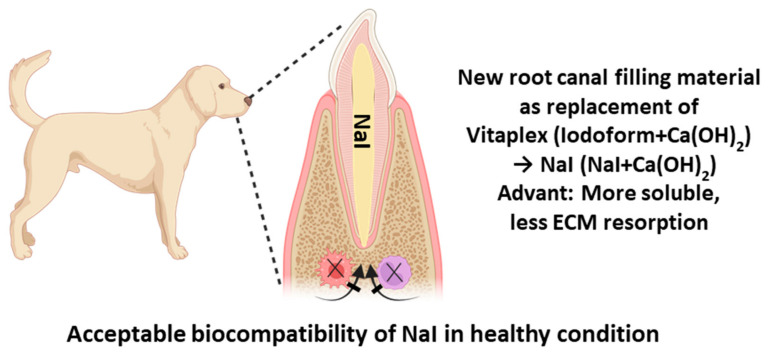
Schematic image of NaI biocompatibility as a root canal filling material in a canine study. Compared to the commercially available calcium hydroxide-based material (Calcipex II) and the calcium hydroxide and iodoform mixture (Vitapex^®^), the newly developed NaI (calcium hydroxide and soluble sodium iodide) showed great biocompatibility as a root canal filling material in healthy teeth after pulpectomy. New root canal filling material (NaI) was developed as a replacement for Vitapex^®^ to overcome the insoluble property of iodoform introduced by sodium iodine (NaI).

This study demonstrates the successful development of a biocompatible sodium iodide-based root canal filling material with an optimized composition that matches the safety profile of current commercial products in healthy pulpectomized teeth (Figure 7). The comprehensive X-ray, CT, histological, and immunological analyses confirmed the material’s biological compatibility in periapical tissues. While the loss of radiopacity remains a consideration for clinical application, the material’s promising safety profile and potential for reduced root resorption warrant further investigation, particularly in infected root canal conditions. Radiopacity could be further enhanced by incorporating biocompatible ZnO_2_ or TiO_2_ particles, though optimization of their concentrations will be necessary to achieve optimal diagnostic visibility while maintaining the material’s other desirable properties. These findings represent a significant step toward developing an improved root canal filling material for primary teeth, though additional research is needed to fully validate its clinical efficacy.

## Figures and Tables

**Table 1:** Quantitative inflammatory score criteria.

Score	Histological Findings	Number of Inflammatory Cells
0	Fibrovascular tissue with mild acute and chronic inflammation	An average of fewer than 10 inflammatory cells observed at 400× magnification
1	Fibrovascular tissue with moderate acute and chronic inflammation	An average of 10 to fewer than 50 inflammatory cells observed at 400× magnification
2	Fibrovascular tissue with severe acute and chronic inflammation	An average of 50 or more inflammatory cells observed at 400× magnification

## Data Availability

The original contributions presented in this study are included in the article and Supplementary Materials. Further inquiries can be directed to the corresponding authors.

## Supplementary Materials

The following supporting information can be downloaded at: https://www.mdpi.com/article/10.3390/ma17246082/s1, Figure S1: Loss of radiopacity of NaI in water; Figure S2: Histological results of positive control *E. faecalis* group.
