# Supplementary material for: Histological Evaluation of Sodium Iodide-Based Root Canal Filling Materials in Canine Teeth
Source: Materials (Basel). 2024 Dec 12;17(24):6082. doi: 10.3390/ma17246082 (PMC11727976; doi:10.3390/ma17246082)
Supplement: Supplementary file 1 [file materials-17-06082-s001.zip › materials-3350463-supplementary.pdf]

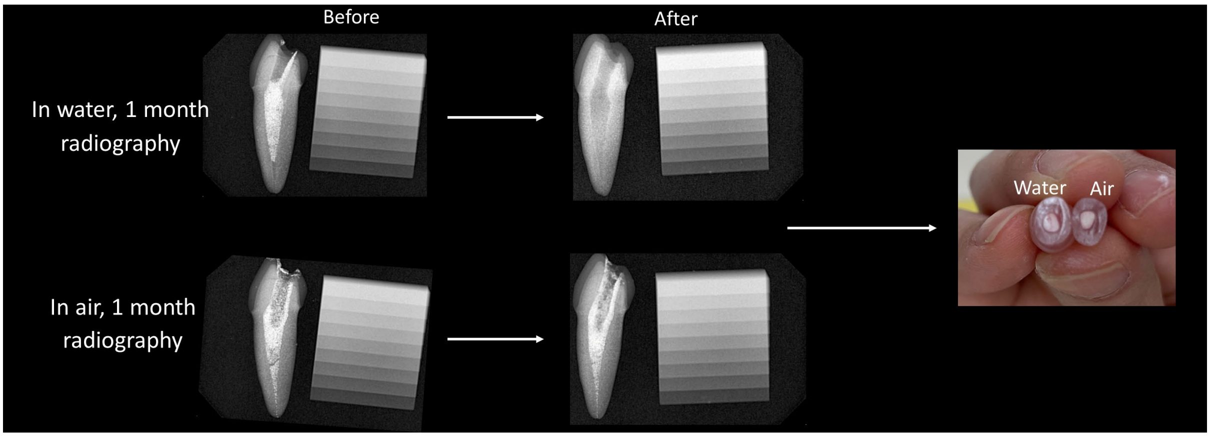

Supplementary Figure S1. Loss of radiopacity of NaI in water. NaI maintained radiopacity in air while losing it during water immersion. Future modification to the material is necessary to maintain radiopacity.

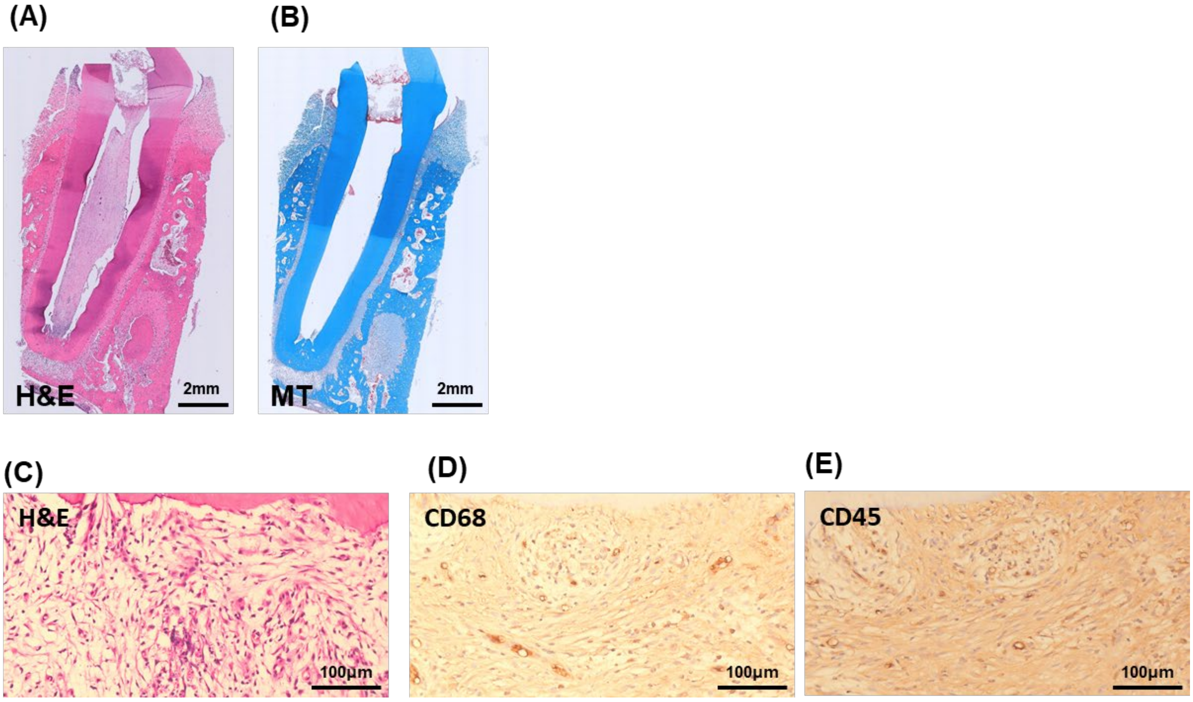

Supplementary Figure S2. Histological results of positive control E. faecalis group. We injected 10 ul of 2.2x10^8 into the pulp chamber after pulpectomy to inflame the periapical lesion. Representative low- and high-magnification histological images are shown (H&E); highly magnified images focus on the periapical regions of mongrel dog teeth after pulpectomy with different root canal filling materials. Immunohistochemical staining was carried out of CD68 and CD45, and a pan macrophage marker and leukocyte marker were used to investigate apical lesion inflammation after applying root canal filling materials, respectively. (**A**) H&E, (**B**) MT, (**C**) high-resolution image of periapical lesion. Immunohistochemical staining of (**D**) CD68 and (**E**) CD45.
